# Monitoring of genetically close Tsaiya duck populations using novel microsatellite markers with high polymorphism

**DOI:** 10.5713/ajas.19.0175

**Published:** 2019-08-26

**Authors:** Fang-Yu Lai, Yi-Ying Chang, Yi-Chen Chen, En-Chung Lin, Hsiu-Chou Liu, Jeng-Fang Huang, Shih-Torng Ding, Pei-Hwa Wang

**Affiliations:** 1 Department of Animal Science and Technology, College of Bioresources and Agriculture, National Taiwan University, Taipei 10672, Taiwan; 2 Ilan Branch, Livestock Research Institute, Council of Agriculture, Executive Yuan, Ilan County 26846, Taiwan; 3 Department of Animal Science and Biotechnology, Tunghai University, Taichung 40704, Taiwan; 4 Livestock Research Institute, Council of Agriculture, Executive Yuan, Tainan 71246, Taiwan

**Keywords:** Breeding and Conservation Strategies, Genetic Structure, Novel Microsatellite Markers, Tsaiya Ducks

## Abstract

**Objective:**

A set of microsatellite markers with high polymorphism from Tsaiya duck were used for the genetic monitoring and genetic structure analysis of Brown and White Tsaiya duck populations in Taiwan.

**Methods:**

The synthetic short tandem repeated probes were used to isolate new microsatellite markers from the genomic DNA of Tsaiya ducks. Eight populations, a total of 566 samples, sourced from Ilan Branch, Livestock Research Institute were genotyped through novel and known markers. The population genetic variables were calculated using optional programs in order to describe and monitor the genetic variability and the genetic structures of these Tsaiya duck populations.

**Results:**

In total 24 primer pairs, including 17 novel microsatellite loci from this study and seven previously known loci, were constructed for the detection of genetic variations in duck populations. The average values for the allele number, the effective number of alleles, the observed heterozygosity, the expected heterozygosity, and the polymorphism information content were 11.29, 5.370, 0.591, 0.746, and 0.708, respectively. The results of analysis of molecular variance and principal component analysis indicated a contracting Brown Tsaiya duck cluster and a spreading White Tsaiya duck cluster. The Brown Tsaiya ducks and the White Tsaiya ducks with Pekin ducks were just split to six clusters and three clusters when K was set equal to 6 and 3 in the Bayesian cluster analysis. The individual phylogenetic tree revealed eight taxa, and each individual was assigned to its own population.

**Conclusion:**

According to our study, the 24 novel microsatellite markers exhibited a high capacity to analyze relationships of inter- and intra-population in those populations with a relatively limited degree of genetic diversity. We suggest that duck farms in Taiwan could use the new (novel) microsatellite set to monitor the genetic characteristics and structures of their Tsaiya duck populations at various intervals in order to ensure quality breeding and conservation strategies.

## INTRODUCTION

The domestic duck was derived from the characteristically green-headed Mallard, *Anas platyrhynchos*, which is widely distributed over the northern hemisphere. Since more than 4,000 years ago, duck domestications have been occurred on many places of worldwide [1]. Asia keeps 87% of ducks of the whole world, with a tremendous variety of duck breeds, such as Shaoxing, Jingdin, Shanma, Liancheng, Bai, and Gaoyou duck in China, Tsaiya in Taiwan [2]. However, the abundant genetic diversity of ducks in Asia is under the pressure of foreign species improvement [3]. Recent, breeding trends in the production of duck in Asian countries seems to pursue higher performance in egg and meat production, and introduced such as Cherry Valley, Pekin and Muscovy. This development definitely will endanger the population of indigenous breeds. Fujihara and Xi [4] pointed out genetic diversity is needed by any species in order to maintain reproductive vitality, resistance to disease, and the ability to adapt to changing conditions in the world. The Tsaiya duck, originally domesticated in China, is the major laying duck in Taiwan, which is one of the best duck egg layers in the world [5]. Before 1970, these ducks exhibited considerable variation in plumage color ranging from solid black to pure white. Due to the farmers’ preference, ducks with light brown plumage were selected and kept as the major variety of Tsaiya. Thus “Brown Tsaiya” became a common name for the local Tsaiya duck [6].

**Figure 1:**
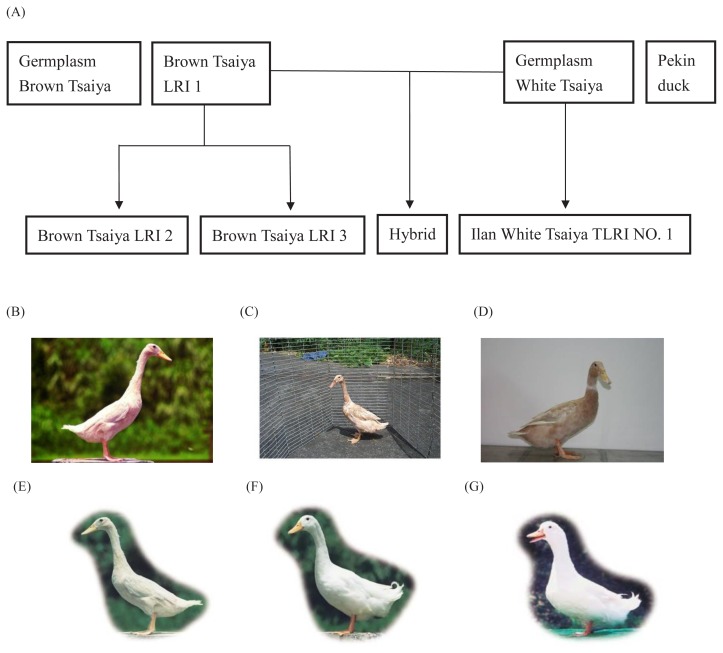
The relation chart and the appearance of each experimental duck breed (line). (A) The breeding original of eight populations in this study. (B) The Brown Tsaiya LRI 1 (BI); (C) The Brown Tsaiya LRI 2 (BII); (D) The Brown Tsaiya LRI 3 (BIII); (E) Germplasm Brown Tsaiya (GBT); (F) Germplasm White Tsaiya (GWT); (G) Pekin duck.

The Livestock Research Institute (LRI), Council of Agriculture, Executive Yuan is the only institute to do research and provide assistance to the duck industry in Taiwan. Genetic selection, improvement and conservation of duck breeds are their major responsibilities. The relationship and characteristics of the eight LRI populations in this study are shown in Figure 1A and described as follows: The Germplasm Brown Tsaiya (GBT) line (Figure 1E) was plotted using native Tsaiya ducks that have been collected by LRI Ilan branch since 1987. The appearances and genetic variety of this duck line have been maintained for fifteenth generations so far through the implementation of a rotational mating system [7]. LRI started a breeding project of Brown Tsaiya in 1984. The selection index for Brown Tsaiya applied at LRI aims at a maximum genetic improvement of egg number at 40 weeks without reducing egg weight or increasing body weight [6]. This high egg production line was namely Brown Tsaiya LRI 1 (BI) (Figure 1B). The second line of LRI’s Brown Tsaiya, that is, the Brown Tsaiya LRI 2 (BII) (Figure 1C), originated from the fifth generation of BI and was selected to increase the number of fertile eggs after a single artificial insemination with pooled Muscovy semen since 1992 [8]. In 1996, LRI started to select the color of the egg shell from the eighth generation of BI and established a Brown Tsaiya line with blue egg shells which Taiwan people favor, called Brown Tsaiya LRI 3 (BIII) (Figure 1D) [5,9]. For removed of black plumage on skin of Mule duck, the selection of White Tsaiya started in 1966. White Tsaiya were collected from duck farms on Taiwan into Ilan branch, LRI and applied in a closed herd to removed colored plumage. After 19 years, 1985, this population was named as Ilan White Tsaiya TLRI NO. 1 [6]. The White Tsaiya of Ilan branch were separated into 3 lines according to the direction selection: L101 as a germplasm preservation stock (Germplasm White Tsaiya, GWT) (Figure 1F) was derived from original population without any selection project. L102, which was named Ilan White Tsaiya TLRI NO. 1 (WI) officially, as a dam line for crossing Muscovy drakes was derived from White Tsaiya control line and continue monitoring and selecting color of feather of mule ducks. L103 was an inbred line by full-sib mating, however this line has been discarded nowadays [6,10]. The Pekin duck (P) is the best known of all table breeds and originated in China and was introduced to the US in 1873. The Pekin duck in Taiwan were transferred from the US in 1954 as three hundred hatching eggs (Figure 1G) [11].

The four new and two original egg-production duck breeds should be maintained and conserved for economic needs in Taiwan. Traditionally, the breeding strategies for a closed population were managed on the basis of visible phenotypic traits. However, the significance of breed is not just their specific identities. The separated genetic composition of each breed is the matter [12]. The information about genetic diversity within and between breeds and population genetic structures is very important to draw the essential outline for any appropriate conservation and sustainable management program. The increasing availability and development of molecular markers techniques is improving our ability to identify the genetic variation of breeds [13]. Six Tsaiya duck breeds, including original and selected, raised and developed in LRI, will keep pure breed raising and preserving. Among these breeds, there were just differences in color or single function. Therefore, the dissimilarity of genetic composition was supposed too limited to differentiate.

Microsatellites, also known as simple sequence repeats (SSRs), represent a class of repetitive sequences widely distributed in all genomes. They are abundant, ubiquitous, easy to automate, codominant, universal, robust, reliable and reproducible markers. Polymorphism patterns exhibited by microsatellites are greater than any other contemporary DNA molecular marker system [14]. In recent years, the polymorphism of short tandem repeats (STR) sequences is increasingly being supplemented with a different type of DNA molecular marker involving single nucleotide polymorphisms (SNPs) in DNA marker. However, SNPs panels have much lower discriminatory power than STR markers. Therefore, SNPs are a supplement for STR markers rather than a replacement [15]. It is comparative overview of SSR and SNP panels for studies of parentage and livestock animal populations of mammals, fishes and birds, the scholars pointed out that the SSR molecular marker has advantages of lower the development cost, lower running cost, lower quality of DNA required, lower quantity of DNA required, and lower technical expertise [14]. Some microsatellite markers have been developed from Tsaiya duck [16]. However, the markers with more polymorphism were needed for Tsaiya duck populations of LRI. In this study, the novelty developed microsatellite markers with high polymorphism were isolated, accompanied with previous markers [16], to analyze and monitor the genetic structure and variation of intra and inter-populations of LRI.

The fitness of Tsaiya duck to their environment is the results of natural selection for centuries in Taiwan. Before replacing them with “improved breeds”, we ought to think about the irreversible loss of genetic diversity or has a certain conservation strategy to be developed in Taiwan. Before formulating these conservation strategies, it is one of the most important missions to clearly understand and define the genetic structure of the population of Tsaiya (strains) currently in Taiwan. The aim of this study was to create and collect a set of microsatellite markers with high polymorphism for the genetic monitoring and genetic structure analysis of Tsaiya (strains) duck populations.

## MATERIALS AND METHODS

### Experiment animals and sample collection

A total of eight populations of LRI were investigated in this study. Six of these populations were Tsaiya ducks. There are two major breeds of Tsaiya duck: White Tsaiya and Brown Tsaiya duck. After a period of selection and breeding, two populations of White Tsaiya and four populations of Brown Tsaiya were raised in LRI, including the GBT (Figure 1E), BI (Figure 1B), BII (Figure 1C), BIII (Figure 1D), Ilan White Tsaiya TLRI NO. 1, L101 (GWT) (Figure 1F) and Ilan White Tsaiya TLRI NO. 1, L102 (WI). Among the populations, the hybrid (H) was the progeny of GWT crossed with BI. The P (Figure 1G) was a breed which was raised by LRI. The population was also investigated in this study as a reference. The experimental protocols used in the present study were approved by the Experimental Animal Care and Use Committee of Ilan Branch, Livestock Research Institute, Council of Agriculture, Executive Yuan, Taiwan.

Blood samples were collected from 566 individuals from these 8 populations, including 43 GBT, 69 BI, 95 BII, 72 BIII, 80 GWT, 72 WI, 96 H, and 39 P individuals. For each bird, 3 mL of blood were drawn from the superficial plantar metatarsal vein or wing vein. Whole genomic DNA was then extracted with Genomic DNA Isolation Reagent (GenePure Technology CO., LTD, Taiwan) using the standard phenol-chloroform method.

### Isolation of microsatellite loci from Tsaiya duck

Genomic DNA was isolated from blood taken from one male and one female Tsaiya duck using a previously described method; the following steps were slightly modified from the procedure previously described by Glenn and Schable [17]. The isolated genomic DNA was partially digested with *Rsa*I and *Xmn*I (NEB, USA) until most of the DNA fragments were between 300–1,000 bp in length. Re-naturing of single strand SuperSNX24 forward (5′GTTTAAGGCCTAGCTAGCAG AATC3′) and SuperSNX24+4P reverse (5′pGATTCTGCTA GCTAGGCCTTAAACAAAA3′) formed SuperSNX24 linkers that were ligated to the digested DNA fragments. Linked DNA fragments were then amplified with SuperSNX24. Next, microsatellite markers including fragments with biotinated probes were isolated and enriched. Three probe compositions were used: i) (TG)_12_, (ACT)_12_, (ACTG)_6_, and (ACAG)_6_; ii) (AG)_12_, (ACAT)_8_, (AACT)_8_, and (AAGT)_8_; and iii) (AAG)_8_, (AAAC)_6_, (AATC)_6_, and (AGAT)_6_. The biotinated probes then were annealed to fragments of gDNA containing complementary regions. Finally, the microsatellite-containing fragments were enriched using streptavidin-labeled metal beads (Dynabeads M-280 Streptavidin, catalog #11205D, Invitrogen, Carisbad, CA, USA). The enriched segments were then cloned into pGEM-T Easy vector (pGEM-T Easy Vector system, Promega, Madison, WI, USA) and sequenced with an ABI PRISM 3730XL DNA Analyzer (Applied Biosystems, Foster City, CA, USA). The fragments with higher repeats were then selected for polymorphism testing.

Seven published microsatellite markers were added to the marker set in this study, including APT004, APT005, APT008, APT015, APT020, APT025, and APT031[16].

### Polymerase chain reaction and polymorphism test

The selected highly repeated fragments were subjected to polymerase chain reaction (PCR) and polymorphism testing which would verify if the microsatellite loci could be amplified and used to show diversity in the investigated populations. Primers for loci amplification were designed using Primer3plus [18]. CAG-tag (5′-CAGTCGGG CGTCATCA-3′) or M13Reverse (5′-GGAAACAGCTATGACCAT-3′) was added to the 5′ end of one of each primer pair [19]. Following the protocol described by Schuelke [20], a fluorescent dye-labeled tag, as a third primer, was used with the primer pair to amplify the target fragments which were detectable on the capillary electrophoresis. Thirty unrelated Tsaiya ducks from the LRI were tested. PCR was performed on a 20 μL volume using a thermalcycler (GeneAmp PCR system 9700, Applied Biosystems, USA) containing 0.5 U *Taq* DNA polymerase (TAKARA, Kyoto, Japan), 1×PCR buffer (1.5 mM MgCl_2_), 0.2 mM dNTP, 0.2 μM unlabeled primer, 0.04 μM tag-labeled primer, 0.16 μM dye-labeled tag, and 50 ng gDNA. The PCR cycling program was as follows: 95°C for 5 min, 35 cycles of 95°C for 30 s, 50°C to 65°C for 40 s, 72°C for 40 s, and a final elongation at 72°C for 7 min. The amplified microsatellite PCR products were analyzed with a DNA analyzer (ABI PRISM 3730 DNA analyzer, Applied Biosystem, USA). Allelic sizes of all loci were estimated relative to in-line GeneScan500 LIZ Size Standard marker (ABI PRISM, Applied Biosystem, USA). The fragment size was calibrated and analyzed with Peak Scanner Software version 1.0 (ABI PRISM, Applied Biosystem, USA). The loci which had an allele number (N_a_) greater than two and similar annealing temperatures were selected for whole population analysis.

### Statistical analysis

For each locus and population and across populations, commonly derived statistics from the microsatellite genotypic data including allele frequencies, the observed number of alleles (No), the observed heterozygosity (H_O_), the expected heterozygosity (H_E_), and the polymorphic information content (PIC) were calculated with the Microsatellite Toolkit. The Hardy-Weinberg equilibrium (HWE) test was performed using the GENEPOP computer program, which also was used to estimate F-statistics (*F*_IT_, *F*_IS_, and *F*_ST_) for each locus, the pairwise *F*_ST_ between populations, and the average inbreeding coefficient (*F*_IS_). Nei’s genetic distance (D_A_) between populations was measured with Microsatellite Analyzer. The phylogenetic tree was generated via the PHYLIP program using the neighbor-joining (NJ) method with bootstrap test of 1,000 resamplings of loci with replacement. The genetic distances of the proportion of shared alleles (POSA) was used to estimate and draw a POSA individual phylogenetic tree [21].

A hierarchical analysis of variance was carried out to allow the partitioning of total genetic variance into components owing to region, population, and individuals. Computations were carried out using a hierarchical analysis of molecular variance (AMOVA) procedure, as implemented in the ARLEQUIN 3.5 package. Principal component analysis (PCA) was performed with GENALEX v.6.501 software in order to spatially plot clusters and individuals based on the distance matrix with data standardization [21].

The model-based approach proposed for population structure analysis of the eight populations was carried out with the software STRUCTURE 2.3.1 [22] which assessed the genomic clustering (K) of the sample. To obtain a representative value of K for data modeling, ten independent runs were performed for each value from one to seven. The run length was set to 100,000 burn-ins following by 100,000 iterations. The ΔK estimated the most likely number of K that represented the population structure [23]. Subsequently, the CLUMPP software 1.1.2 [24] was used to demonstrate the optimal alignment of the 20 replicates for the same K value. The mean membership matrix across replicates was calculated with DISTRUCT v.1.1 [25].

## RESULTS

### Isolation of novel microsatellite Loci

Over 50 DNA fragments containing STRs were cloned. A polymorphism test was performed using the LRI populations, and 17 loci which had allele numbers more than two were picked. The 17 novel markers included three complex repeats (T1P4017, T3P1027, and T3P2090). In simple repeat markers, there was one dinucleotide unit (T1P1075), and four trinucleotide units (TYD037, TYD038, TYD042, and T1P2013), while others contained tetranucleotide units. These novel markers were then combined with the seven previously known markers [16], and together formed a set of markers for this study (Table 1).

### Polymorphism, heterozygosity, and *F*-statistics of selected microsatellite loci

Polymorphism was clearly observed at all the microsatellite loci in all eight of the LRI duck populations. The genetic characteristics of the 24 microsatellite loci are listed in Table 2. The average number of alleles per locus (N_a_) was 11.292. The actual number of alleles ranged from 2 (T3P1027) to 36 (TYD029). The average number of effective alleles per locus (N_e_) ranged from 1.816 (T3P2090) to 16.588 (TYD029), with an average across loci of 5.370. The PIC value ranged from 0.375 (T3P1027) to 0.937 (TYD029), with an overall average of 0.708. All of the selected microsatellite loci in this study were sufficiently polymorphic, indicating that these loci were suitable for the genetic analysis of ducks.

The HE among the 24 microsatellite loci had a range of 0.450 (T3P2090) to 0.941 (TYD029), with the average value of H_E_ being 0.746. The H_O_ among the 24 microsatellite loci had a range of 0.347 (T3P2080) to 0.808 (TYD024), with the average value of H_O_ being 0.591 (Table 2). However, there were four loci, namely, TYD021, T1P2013, T3P2090, and APT004, that significantly departed from the HWE (p<0.01).

The Wright’s *F*-statistic values (*F*_IS_, *F*_IT_, and *F*_ST_) for each locus are shown in Table 2. The average *F*_IS_ for all the loci was 0.043, and the *F*_IS_ per locus varied from −0.093 (APT020) to 0.304 (TYD025). The average *F*_IT_ for all the loci was 0.233, and the *F*_IT_ per locus varied from 0.095 (T1P1075) to 0.397 (TYD006). The mean *F*_ST_ for all the loci was 0.197. This value implied that around 19.7% of the total genetic variation was caused by population differences and that 80.3% of total genetic variation was due to genetic differentiation among individuals within each population.

### Intra–population genetic variability and Hardy-Weinberg equilibrium test

The genetic statistics relating to polymorphism, including H_E_, H_O_, PIC, the mean observed number of alleles, and the mean effective number of alleles (N_e_), were calculated to estimate the allelic diversity at each locus of population. These genetic parameters across the 24 loci for the eight duck populations are listed in Table 3. H_E_ varied from 0.535 (WI) to 0.686 (BIII), whereas H_O_ varied from 0.482 (WI) to 0.707 (H) and PIC ranged from 0.461 (WI) to 0.633 (BIII). The WI population had the lowest values of H_O_, H_E_, and PIC. The BIII population exhibited the highest values of H_E_, and PIC. The H population exhibited the highest values of H_O_, and the smallest value of *F*_IS_ (Table 2).

Among the eight populations, the P population had the highest observed mean number of alleles (MNA) (6.542), followed by the hybrid (6.500) and BIII (5.833) populations, while the WI population had the smallest observed MNA (3.708). Negative *F*_IS_ values were observed only in one population (H), indicating an insufficient degree of inbreeding. The deviation from the Hardy-Weinberg proportions within populations (*F*_IS_) varied from −0.047 to 0.103. The highest inbreeding effects were found in the BI population (0.103). The H population contained 16 loci that were significantly deviated from the HWE (p<0.01) (Table 3), and were much higher than other populations.

### Inter-population genetic variation

The values of *F*_ST_ and D for each test population pair are summarized in Table 4. The *F*_ST_ for each population pair was highly significant (p<0.05) The *F*_ST_ values of the population pairs were varied from 0.083 (for the BI and H population pair) to 0.322 (for the WI and BII population pair). There were several pairs of population have sub-lowest *F*_ST_ of which around 0.09 (BI and GBT pair, BI and BIII pair, H and BIII pair and H and GWT pair).

The genetic distances between the duck population pairs varied from 0.157 (for the BI and H population pair) to 0.582 (for the BII and WI population pair). There were relatively high genetic distance among the white duck populations (GWT, WI, and P) and between the other populations (p>0.3) except for GWT and H pair while GWT was paternal of H.

### Population differentiation analysis

**Figure 2:**
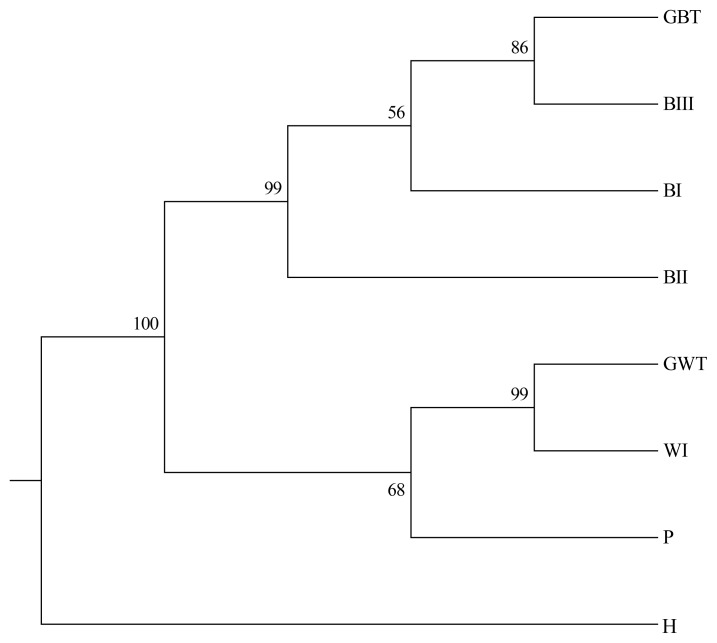
The unweighted pair group method was used with a neighbor joining (NJ) dendrogram summarizing genetic relationships among the eight duck populations based on Nei’s D_A_ distances for the 24 microsatellite loci. The numbers on the nodes indicate the percentage bootstrap values generated from 1,000 re-samplings. The abbreviations of the population names are follows as: GBT, Germplasm Brown Tsaiya duck; BI, Brown Tsaiya LRI 1; BII, Brown Tsaiya LRI 2; BIII, Brown Tsaiya LRI 3; GWT, Germplasm White Tsaiya duck; WI, Ilan White Tsaiya TLRI NO. 1, L102; H, Hybrid of Germplasm White Tsaiya duck×Brown Tsaiya LRI 1; P, Pekin duck.

In this study, the results of the differentiation of the eight duck populations using the NJ tree are shown in Figure 2. In the NJ tree, the brown duck populations (GBT, BI, BII, and BIII) and the white duck populations (GWT, WI, and P) were independently grouped into two clads. The closest relationship in brown duck group was GBT and BIII. The following population was BI, but their bootstrap confidence value was only 56%. The furthermost population among brown ducks were BII. In the white duck clad, P located apart from GWT and WI, of which bootstrap value was 68%. The H populations were depicted as independent taxa.

**Figure 3:**
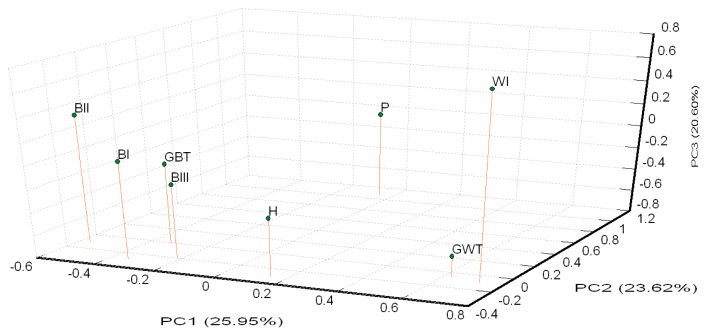
Principle coordinate analysis (PCA) plot of eight population positions by population genetic distances based on the allele frequencies of 24 microsatellite markers (loci). The first (PC1), second (PC2), and third (PC3) principal components accounted for 25.95%, 23.62%, and 20.60% of the total variation, respectively. The abbreviations of the population names are follows: GBT, Germplasm Brown Tsaiya duck; BI, Brown Tsaiya LRI 1; BII, Brown Tsaiya LRI 2; BIII, Brown Tsaiya LRI 3; GWT, Germplasm White Tsaiya duck; WI, Ilan White Tsaiya TLRI NO. 1, L102; H, Hybrid of Germplasm White Tsaiya duck×Brown Tsaiya LRI 1; P, Pekin duck.

A PCA of pair-wise genetic distances among the eight examined goose populations was used to represent the relative positions of the populations. The first (PC1), second (PC2), and third (PC3) principal components accounted for 25.95%, 23.62%, and 20.60% of the total variation, respectively (Figure 3). The results of the PCA were similar to the phylogenetic tree drawn up via the NJ method except that GWT and WI was separated in PC3.

### Population structure analysis

The results of the degree of variance in the duck populations via AMOVA are summarized in Table 5. In the analysis of all the populations, the largest variation was found within individuals (76%), followed by variation among populations (19%). The variation among individuals within populations accounted for 5% of the total variation. In the Brown Tsaiya duck populations, the variation among individuals within the populations accounted for 13% of the total variation, while that among populations accounted for 7% of the total variation. In the White Tsaiya duck populations, the variation within individuals decreased to 70% while that among populations reached to 24%, while the variation among populations in Brown Tsaiya duck populations was only 13%.

**Figure 4:**
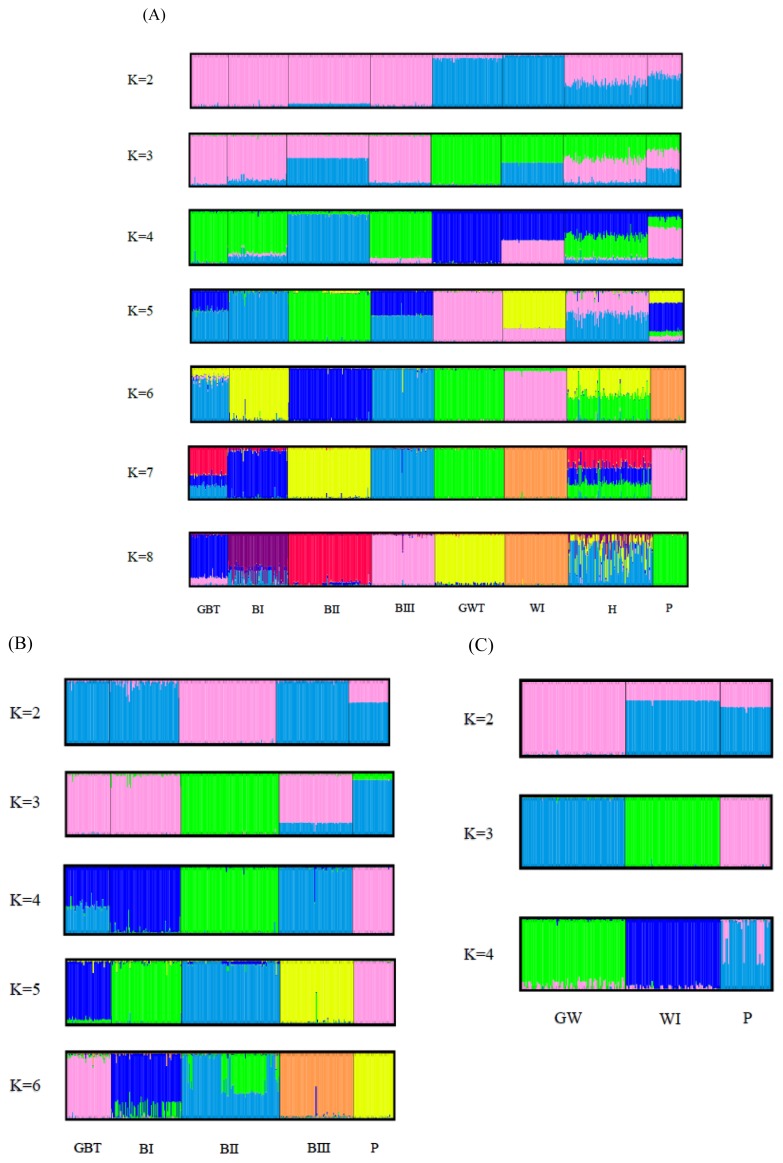
Structural analyses results for the duck populations. Each genotyped duck is represented by a single vertical line divided into K colors, where K is the number of clusters assumed in each structure analysis. Each vertical bar represents an individual duck. The colors on a vertical bar represent the probability that an individual belongs to that cluster. (A) Cluster results from a structural analysis of 566 ducks from eight populations (based on 24 microsatellite markers). Even though K>8, there was no new cluster appearance and the figures were not too different. (B) Clustering analyses of Brown Tsaiya ducks and Pekin ducks. (C) Clustering analyses of White Tsaiya duck and Pekin duck populations. The abbreviations of the population names are follows: GBT, Germplasm Brown Tsaiya duck; BI, Brown Tsaiya LRI 1; BII, Brown Tsaiya LRI 2; BIII, Brown Tsaiya LRI 3; GWT, Germplasm White Tsaiya duck; WI, Ilan White Tsaiya TLRI NO. 1, L102; H, Hybrid of Germplasm White Tsaiya duck×Brown Tsaiya LRI 1; P, Pekin duck.

The results of the STRUCTRE program analysis of all the populations are shown in Figure 4A. White Tsaiya and Brown Tsaiya were definitively separated into different clusters just at K = 2, while H and P became mixed groups. At K = 3, the GWT population was split into a new cluster. At K = 4, the BII population formed another new cluster. All populations were assigned into their cluster except that GBT bound to BII and H just has half BI and half GWT while K was set at six. Seven population excluding H were almost have their clusters until K = 8. No new cluster appeared, however, and the figures were not much different for values of K larger than 8. In order to survey the structures on intra-clad basis accurately, analyses of the white ducks and brown ducks, respectively, were carried out (Figure 4B, 4C). Among the brown ducks and P, the BII population became a subgroups first at K = 2, while the P population subsequently became a new subpopulation at K = 3. Four separately clusters were form at K = 4, that only GBT was a mixed population with the components of BI and BIII. At K = 5, the brown ducks and P were split into 5 clusters exactly. BII population tended to have two subgroups at K = 6. The white ducks were relatively simple. At K = 2, GWT formed a completed cluster and WI and P were the similar mixed clusters. The white ducks were split into 3 clusters exactly at K = 3. When K was over 4, the components of P had more complicated than GWT and WI.

## DISCUSSION

In this study, we used the 24 higher polymorphism microsatellite markers (loci) to test the genetic structure of the eight duck populations. The results showed that the average of N_a_ among known loci was 9.71±2.43, and the average of N_a_ among novel loci was 11.94±9.20. Although the average of N_a_ among novel loci was larger than known loci, the deviation of N_a_ of novel loci was much large (N_a_ was 2 to 36). Among those loci, a number of hypervariable loci with more than 20 alleles in the LRI populations, including TYD005, TYD024, and TYD029, were found. Such complex and hypervariable loci have previously been found in human CODIS loci and some canine microsatellite loci [26] as well. These complex loci might be beneficial for individual differentiation due to their excellent diversity. The P_(ID)_ (probability of identity) values [27] of TYD005, TYD024, and TYD029 were 0.040, 0.040, and 0.048, respectively in the BI population, and their combined value was 7.68×10^−5^, which means these three markers alone could be used to differentiate more than ten thousand individuals.

**Figure 5:**
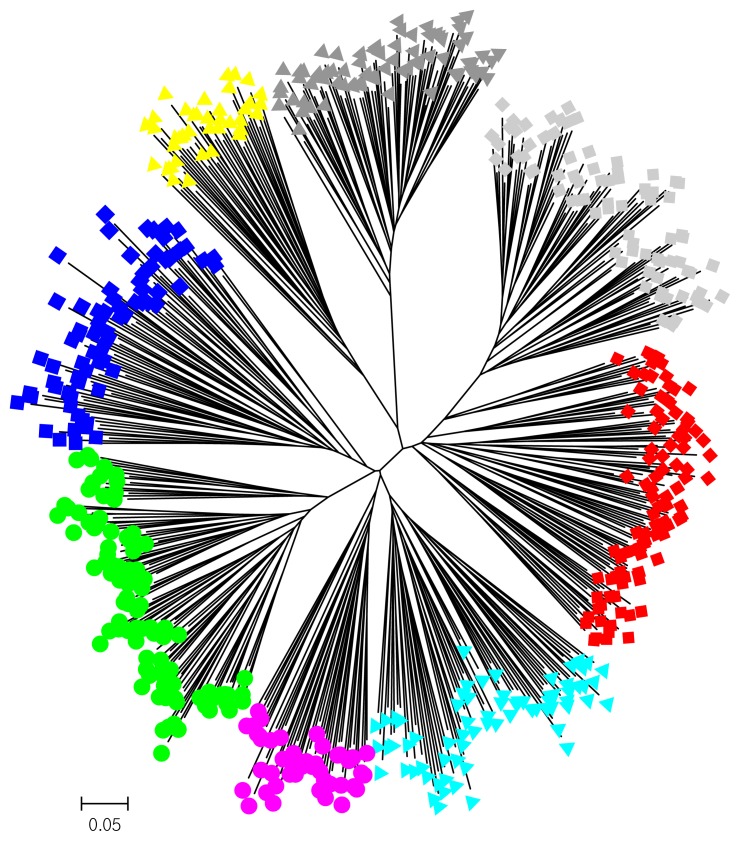
The unrooted individual phylogenetic tree of eight duck populations constructed from –ln (shared allele proportion) by 24 microsatellite marker polymorphisms. 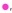, GBT; 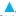, BI; 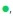, BII; 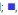, BIII; 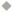, GWT; 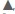, WI; 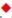, H; 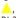, P. The abbreviations of the population names are follows as: GBT, Germplasm Brown Tsaiya duck; BI, Brown Tsaiya LRI 1; BII, Brown Tsaiya LRI 2; BIII, Brown Tsaiya LRI 3; GWT, Germplasm White Tsaiya duck; WI, Ilan White Tsaiya TLRI NO. 1, L102; H, Hybrid of Germplasm White Tsaiya duck×Brown Tsaiya LRI 1; P, Pekin duck.

To examine the diversity of the 17 novel microsatellite makers and the seven published markers in Taiwan Tsaiya ducks, several indicators were calculated from randomly selected samples from the LRI. The H_O_ values of all the markers were higher than 0.3. A previous report suggested that microsatellite markers used in studies of genetic variation and distance should have H_O_ values of between 0.3 and 0.8 in the population [28]. The N_a_, N_e_, and PIC values across all the loci were 11.29, 5.370, and 0.708, respectively, indicating higher genetic variability and diversity in the investigated ducks than among domestic ducks from other areas [29–31]. Significant deviation from the HWE was observed in only four of the 24 loci. By the STRUCTURE, the components in H population were just half of GWT and half of BI (Figure 4A), which the parents of H, at K = 6. Also, an un-rooted individual phylogenetic tree (Figure 5) showed that most of the individuals had been sorted to their population distinctly and that the H population was located just between the GWT and BI. These results indicated that the compilation of all the microsatellite markers used in this study were suitable for evaluating the genetic structures of Tsaiya duck populations in Taiwan.

### Intra-population genetic variation and diversity

In Table 3, the results present the intra-population genetic variation in eight of the Tsaiya duck populations. Deficiencies of heterozygosity (that is, H_O_ lower than H_E_) were exhibited by almost all the populations. Only the H population had excess heterozygosity, while the GBT and GWT populations each had roughly equal H_O_ and H_E_ values. The sources of heterozygous deficit probably included inbreeding, null allele, aneuploidy, misscoring and Wahlund effect [32]. There were all small and closed populations in LRI, however, all populations but GWT and GBT were established by selection of specific traits. GWT and GBT were derived from the original populations without any major selection since before 1980 [7]. The major causes of deficiencies of heterozygosity perhaps were selection of specific traits that accompanied specific alleles. The BII population tended to split into two Brown Tsaiya subpopulations when K = 6 (Figure 4B). The deficiencies of heterozygosity on BII maybe combined a Wahlund effect. The first generation of BI crossed GWT have some characters predictably: the excess heterozygosity (H_E_ = 0.676, H_O_ = 0.707), far from inbreeding (*F*_IS_ = −0.047) and departure HWE (16 of 24 markers HWE departure). In general, except for H, every Tsaiya duck population in LRI was a relatively low *F*_IS_ (almost lower than 0.1), and the numbers of loci departing from the HWE were less than 7. It can thus be concluded that the Tsaiya ducks in LRI probably have good breeding programs. The results of the intra-population diversity analyses for the Pekin populations from the LRI were similar, including relatively high variation (H_O_ = 0.561, PIC = 0.589 and the highest observed MNA = 6.542) and *F*_IS_ values of 0.08. WI was selected for crossing Muscovy drakes to improve the carcass appearances of mule ducks. WI had related low diversity (PIC = 0.461; H_O_ = 0.482) and little more inbreeding (*F*_IS_ = 0.098). This was probably caused by the multiple and long-term selection.

### Inter-population genetic diversity and relationships

Brown Tsaiya and White Tsaiya ducks are the two major domestic breeds in this study, in addition of P and a hybrid population. According to the AMOVA results (Table 5), the proportion of genetic variation attributed to population differences in all populations was about 19%, while the proportion of genetic variation attributed to individuals within populations was 5% of the total genetic variation. Compared to other researches, differentiation was analyzed with 15 microsatellite loci in 26 breeds of Chinese indigenous ducks, and 12.43% of total genetic variation was attributed to differences among populations [33]; four breeds of Indian indigenous ducks were analyzed with 24 microsatellite loci, and 19.76% of the total genetic variation was attributed to differences among populations [34]. However, the Tsaiya populations of LRI were derived from unique species: Brown Tsaiya ducks. The formation of these breeds was just a series of selections and different periods of breeding. The differentiation among populations of Tsaiya ducks in LRI was larger than many different breeds in China and India. The only inference was that higher variance of markers were used in this study [35]. In addition. The variation attributed to individuals within populations was 5%. The high inter-population and low intra-population variations was very benefit to discriminate the different populations that even had similar appearances. The individual phylogenetic tree (Figure 5) revealed that not only separated entirely eight populations, but the individuals were assigned exactly to their own population. Although all Brown Tsaiya populations were very close in PCA (Figure 3). Such high resolution, the 24 microsatellite loci became an excellent tool to monitor the genetic structure of each Tsaiya ducks population, and to consult a breeding program for breed conservation. When AMOVA analysis focused on Tsaiya ducks (six populations), the variance among populations (22%) were comparatively more heterogeneous than all populations (19%), and phenomenon which might have been caused by the differences between the Brown and White Tsaiya ducks. This hypothesis was confirmed with the drawing of the NJ phylogenetic tree, which showed two major clads of brown and white (including P) ducks (Figure 2), and through the STRUCTURE analysis, which revealed that the brown and white populations were divided into two parts when K = 2, with the H population split into two contributions (Figure 4A). The PCA plot showed that the brown and white ducks were significantly separated in terms of PC1, while the hybrid ducks were located between them (Figure 3). The individual phylogenetic tree (Figure 5) also divided the brown and white ducks into two clusters, with the hybrid ducks and P inserted between them. In our distinct analyses, the H population was always situated between two major groups or bisected individuals at K = 2 to K = 6, especially two parts just composed GWT and BI while almost populations were assigned to specific clusters. The 24 microsatellite markers used in this study have the ability to detect any crosses between two breeds.

The Brown Tsaiya populations in LRI were originated from GBT population and selected and derived to BI population, then BII and BIII populations were derived from BI. Moreover, LRI have been bred these four populations and have been kept their characters for becoming a breed. During such period, Monitoring their genetic characteristics and structures was very important. In AMOVA, the proportion of genetic variation of brown duck populations that came from population differences was only 13%, less than all Tsaiya duck populations, 22%. The four populations were close and compact as a group that far from other populations in 3D PCA. It’s perhaps all BI, BII, and BIII were derived from GBT and all were selected by only one trait, moreover their separation was not for long time, the selective programs began at 1984, 1992, and 1996 respectively [6,8,9]. Such low diversity between populations and each population would breed for a breed, the monitoring and analyzing system should be high definition. The unrooted individual phylogenetic tree (Figure 5) showed that four Brown Tsaiya duck populations were close but parted. The STRUCTURE analysis of the Brown Tsaiya ducks (Figure 4B) also revealed that every individual that belonged to the same line was simply assigned to the same population, even when the portions in an individual were almost close to 100%. Our microsatellite markers had high definition and were suitable for analyzing the genetically close populations. Unexpectedly, the Brown Tsaiya and P populations were not isolated until K = 3. BII was isolated first at K = 2. However, P located on different clad of Brown Tsaiya ducks in NJ phylogenetic tree and separated from Brown Tsaiya duck cluster at PC2 in PCA. In our analysis, P still was a very different breed from Tsaiya duck. As the expectation, BII was split to a specific group first at K = 2, matching the results of neighbor joining phylogenetic tree and PCA that BII was a peripheral group of Brown Tsaiya ducks. It was maybe that BII raised and bred on Tainan, south of Taiwan, nevertheless, other Brown Tsaiya duck populations were on Ilan branch, north of Taiwan. The geographic separation and continuous selection [9] probably were the causes of this phenomenon. As BII, BIII was selected from BI, but its variation or distance to GBT was even less than BI’s. It could be caused by that genetic structure of BIII, picked with blue shell eggs, was closer to GBT. Otherwise, BIII and GBT have the highest H_O_ (BIII = 0.631, GBT = 0.661) and PIC (BIII = 0.633, GBT = 0.623) (Table 3). High polymorphism perhaps make both of them similar genetic content. The BII population was even split into two clusters at K = 6 (Figure 4B). Selection in a closed population may have been the cause of this.

In contrast, belonging a clad in phylogenetic tree, the white duck populations (including P) were dispersal in PCA than brown ducks, that WI was split away from GWT at PC3 (Figure 3). The separating far away their co-original population maybe was caused by the progeny test for the white hybrid mule that needed long term selection. The diversity of WI was lost during the breeding procedure that was expressed in the lowest H_O_ (0.482) and observed MNA (3.708) (Table 3). Chang et al [10] reported the same trend that there were just three clearly distinguishable populations at K = 3, and that even at K = 4, two individual White Tsaiya ducks were still assigned fully to their own population (Figure 4C). It showed that two white ducks populations were totally differentiation under analysis of our microsatellite markers. On the other hand, P had split into two sub-populations while K = 4.

## CONCLUSION

The present study verified that the 24 high polymorphic microsatellite loci, including the 17 novel loci isolated in this study, were useful in studying the relationships and genetic diversities among the Tsaiya duck populations in LRI even there were almost similar genetically content. In addition, these markers could be applied continually in genetic monitoring for quality analysis and breeding of ducks in Taiwan. Analysis through 24 microsatellite markers, the intra-population with high diversity and low *F*_IS_, inter-population with low genetic distance and POSA or model-based approach with proper assigning and almost single portion individuals indicated the breeding conditions of Tsaiya ducks in LRI is generally fit demand. At last, the suggestion was that LRI and other duck farms in Taiwan could use the new microsatellite set to genetic monitor their Tsaiya populations at intervals for quality breeding and conservation strategies.

## Figures and Tables

**Table 1:** Primer sequences, repeated motifs, fluorescent labeling, and annealing temperatures of 24 microsatellite loci

Locus	Primer sequence (5′ - 3′)	Repeat motif	Size (bp)	Annealing Temp. (°C)
TYD005	F: ACATGGGGAGAAGAGGATCA	(TCCA)_41_	137–255	
	R: GTTCTGACTCAGGGGATGGA			58
TYD006	F: ATCACAGGCTCAGAGCACCT	(TCCA)_34_	104–208	58
	R: CCTCAGCAGGTCTAGGTTGG			
TYD012	F: GTCATCGTGCAGGTGAGGA	(TGGA)_35_	151–227	58
	R: CTGAAACCTGGAGGCTGTGT			
TYD015	F: TGCTGAAATGGTCAACATCC	(TGGA)_25_	200–250	
	R: TCTTCTATCTCCTCCCCCTGA			58
TYD021	F: TCAGAATACTGATTGCATTTGTCC	(CAAA)_13_	212–220	58
	R: TGCTGCATAAAGACACGGAG			
TYD024	F: GGGAAGGAAAGGTGAGAAGG	(CTTT)_31_	260–392	58
	R: GACGGAAGGACGAAATAAAGG			
TYD025	F: TGATTGCCTGATAGTCATCTTGA	(CTTT)_31_	264–342	58
	R: CCACCTTTAATCGTTGCTACA			
TYD029	F: GATTGTTGGTTTCAACAAATGG	(CTTT)_40_	298–387	
	R: ACGAAAACAGGAACCAAACG			58
TYD037	F: CACACTGCAATGCACCAAAT	(AAG)_53_	251–323	58
	R: TTGTTCACAACAAACTTCAGGA			
TYD038	F: TTGTTGCTGGCTAACAGTGC	(AAG)_69_	246–378	58
	R: TCTTAGCCATTTGCCTTTCC			
TYD042	F: CCACAGCTCTCCGAAATCC	(GCT)_40_	404–438	58
	R: GCTCCTTCCCTTGCAGATTA			
T1P2013	F: AAGTGCTGAGGTCTTGAGAG	(AAG)_53_	270–336	60
	R: CCACATCTCAGGTATCTTGGC			
T1P4017	F: TCCAGCACTAGTACATGCTAAG	(AAAC)_4_... (AAC)_6_	413–422	60
	R: CGGTATGGAGGGCATACAATC			
T3P1027	F: CTTTACAAGGGAGCAGGCAC	(AC)_7_... (AAAC)_4_	333–335	60
	R: TGGTCCTGCATCCTCTCTTC			
T3P2090	F: CCTTTATCAGTAGGGTCAGCC	(AAAC)_5_...(AC)_7_	425–430	60
	R: GCACTAACTTCGGCACACAG			
T1P1075	F: TTGTCAGCCTCCCTCATTCC	(AC)_8_	295–299	60
	R: GGTGTGTTTCGCTGTCAG			
T3P2080	F: TCTCATGAGGCCAGTGTCAC	(AAAC)_8_	394–402	60
	R: GCATGCATGTAACTCGAAGAC			
APT0041)	F: GGGCAGGAAAATCTCCTGAAT	GATAGAT(GATA)_15_	280–316	57
	R: TCTCAGTGGCTGAGCGGTC			
APT005	F: TCCGTACAGACCAACATCGG	(GATA)_17_	281–321	57
	R: AGGTCTTTACAGCCCACTCCC			
APT008	F: CAAAGAAATCCTAGAACATCATTCAAAT	(GATA)_12_	178–198	57
	R: TCTTCTGGCTTTTCACCTTAGTTTAGTA			
APT015	F: CTGTTATGACACCATGTTTGGATTTA	(GATA)_13_	124–161	57
	R: CGTGCTCTGCAACAACTGAAA			
APT020	F: TTCCAAGTTTGTCATGCCAATAGA	(GATA)_14_	137–200	57
	R: CTGACCATGTTAGGGCGTTTTAG			
APT025	F: TCCTAAGAAACGTTGCTTCATAGACC	(GATA)_13_	102–132	57
	R: GAGTTAAGCTTCATCACTCTGTGACTG			
APT031	F: GCTGGAAGAAAGGAGAAGGAGG	(GATA)_12_	185–237	57
	R: AGAAAAACAGTATGAGCGAACAGGT			

1) APT004, APT005, APT008, APT015, APT020, APT025, and APT031 from Hsiao et al [16].

**Table 2:** Characteristics of 24 microsatellite markers used in eight duck populations of The Livestock Research Institute

Locus	*F*_IS_	*F*_ST_	*F*_IT_	N_a_	N_e_	H_O_	H_E_	PIC	Exact test of HWE
TYD005	0.204	0.212	0.373	21	8.972	0.601	0.889	0.878	NS
TYD006	0.296	0.143	0.397	14	4.556	0.480	0.781	0.754	NS
TYD012	0.128	0.180	0.286	11	5.725	0.633	0.826	0.803	NS
TYD015	0.103	0.280	0.354	16	6.117	0.585	0.837	0.819	NS
TYD021	−0.071	0.224	0.169	4	2.979	0.572	0.665	0.603	*
TYD024	0.004	0.147	0.150	23	11.303	0.808	0.912	0.905	NS
TYD025	0.304	0.115	0.384	19	7.139	0.541	0.861	0.845	NS
TYD029	0.073	0.147	0.209	36	16.588	0.764	0.941	0.937	NS
TYD037	−0.049	0.298	0.264	9	3.885	0.579	0.743	0.705	NS
TYD038	0.035	0.141	0.171	13	3.204	0.585	0.688	0.637	NS
TYD042	−0.042	0.208	0.174	5	2.099	0.447	0.524	0.433	NS
T1P2013	0.059	0.182	0.230	15	8.283	0.698	0.880	0.868	*
T1P4017	−0.055	0.186	0.141	5	3.409	0.584	0.707	0.658	NS
T3P1027	0.068	0.179	0.235	2	1.999	0.383	0.500	0.375	NS
T3P2090	−0.014	0.224	0.213	3	1.816	0.367	0.450	0.390	*
T1P1075	−0.012	0.105	0.095	3	2.361	0.531	0.577	0.510	NS
T3P2080	0.151	0.275	0.385	4	2.137	0.347	0.532	0.480	NS
APT0041)	0.038	0.143	0.175	10	6.545	0.718	0.848	0.828	*
APT005	−0.044	0.225	0.191	11	5.958	0.695	0.833	0.811	NS
APT008	0.014	0.173	0.184	6	3.879	0.635	0.743	0.706	NS
APT015	−0.028	0.227	0.205	10	4.615	0.642	0.784	0.750	NS
APT020	−0.093	0.209	0.135	11	5.104	0.722	0.805	0.775	NS
APT025	−0.072	0.257	0.204	7	3.862	0.617	0.742	0.701	NS
APT031	0.024	0.246	0.264	13	6.345	0.658	0.843	0.823	NS
Mean	0.043	0.197	0.233	11.292	5.370	0.591	0.747	0.708	-

*F*_IS_, the measure of the deviation from the Hardy-Weinberg proportions within subpopulation; *F*_ST_, the measure of the degree of differentiation between subpopulations; *F*_IT_, the measure of the deviation from the Hardy-Weinberg model for the total population; N_a_, number of observed alleles; N_e_, effective alleles; H_O_, observed heterozygosity, H_E_, expected heterozygosity; PIC, polymorphism information content; HWE, Hardy-Weinberg equilibrium; NS, not significant.

* Significant (p<0.01) departure from the Hardy-Weinberg equilibrium.

1) APT004, APT005, APT008, APT015, APT020, APT025, and APT031 from Hsiao et al [16].

**Table 3:** Genetic parameters across 24 loci in the eight duck populations

Population1)	*F*_IS_	PIC	Mean heterozygosity		MNA		Number of loci departure from HWE
	
			Expected (H_E_)	Observed (H_O_)	Effective	Observed	
GBT	0.039	0.623	0.681	0.661	3.583	5.792	3
BI	0.103	0.595	0.645	0.584	3.486	5.667	6
BII	0.057	0.507	0.558	0.527	2.781	5.083	4
BIII	0.079	0.633	0.686	0.631	3.782	5.833	7
GWT	0.032	0.495	0.559	0.541	2.518	4.500	6
WI	0.098	0.461	0.535	0.482	2.259	3.708	5
H	−0.047	0.629	0.676	0.707	3.905	6.500	16
P	0.080	0.589	0.639	0.561	3.580	6.542	6

*F*_IS_, the measure of the deviation from the Hardy-Weinberg proportions within a subpopulation; PIC, polymorphism information content; MNA, mean number of alleles; HWE, Hardy-Weinberg equilibrium.

1) GBT, Germplasm Brown Tsaiya duck; BI, Brown Tsaiya LRI 1; BII, Brown Tsaiya LRI 2; BIII, Brown Tsaiya LRI 3; GWT, Germplasm White Tsaiya duck; WI, Ilan White Tsaiya TLRI NO. 1, L102; H, Hybrid of Germplasm White Tsaiya duck×Brown Tsaiya LRI 1; P, Pekin duck.

**Table 4:** Pair-wise estimates of breed differentiation (*F*_ST_) (below the diagonal) and genetic distance (D) (above the diagonal) between each pair of the eight duck populations

Population	GBT	BI	BII	BIII	GWT	WI	H	P
GBT	-	0.175	0.252	0.195	0.469	0.535	0.254	0.450
BI	0.092*	-	0.199	0.211	0.514	0.503	0.157	0.502
BII	0.167*	0.148*	-	0.294	0.471	0.582	0.253	0.499
BIII	0.093*	0.103*	0.162*	-	0.495	0.487	0.283	0.469
GWT	0.241*	0.258*	0.297*	0.198*	-	0.360	0.171	0.458
WI	0.285*	0.287*	0.322*	0.234*	0.240*	-	0.339	0.575
H	0.115*	0.083*	0.167*	0.090*	0.091*	0.214*	-	0.424
P	0.190*	0.200*	0.247*	0.193*	0.257*	0.286*	0.179*	-

GBT, Germplasm Brown Tsaiya duck; BI, Brown Tsaiya LRI 1; BII, Brown Tsaiya LRI 2; BIII, Brown Tsaiya LRI 3; GWT, Germplasm White Tsaiya duck; WI, Ilan White Tsaiya TLRI NO. 1, L102; H, Hybrid of Germplasm White Tsaiya duck×Brown Tsaiya LRI 1; P, Pekin duck.

* Pairwise FST was significant at p<0.05.

**Table 5:** Hierarchical analysis of molecular variance within and among populations of Tsaiya duck

Sample	Number of populations	Variance of components (%)		

		Within individuals	Among individuals within populations	Among populations
All	8	76	5	19
Tsaiya ducks	6	72	6	22
Brown Tsaiya ducks	4	80	7	13
White Tsaiya ducks	2	70	6	24
